# Crystal structures of four dimeric manganese(II) bromide coordination complexes with various derivatives of pyridine *N*-oxide

**DOI:** 10.1107/S2056989019010557

**Published:** 2019-07-30

**Authors:** Sheridan Lynch, Genevieve Lynch, Will E. Lynch, Clifford W. Padgett

**Affiliations:** a Georgia Southern University, 11935 Abercorn St., Department of Chemistry and Biochemistry, Savannah GA 31419, USA

**Keywords:** crystal structure, manganese(II) bromide, pyridine *N*-oxide ligand, complex, offset π-stacking, hydrogen bonding

## Abstract

Four manganese(II) bromide coordination complexes have been prepared with four pyridine *N*-oxides, *viz*. pyridine *N*-oxide (PNO), 2-methyl­pyridine *N*-oxide (2MePNO), 3-methyl­pyridine *N*-oxide (3MePNO), and 4-methyl­pyridine *N*-oxide (4MePNO). The compounds are bis­(μ-pyridine *N*-oxide)bis­[aqua­dibromido­(pyridine *N*-oxide)manganese(II)], [Mn_2_Br_4_(C_5_H_5_NO)_4_(H_2_O)_2_] (**I**), bis­(μ-2-methyl­pyridine *N*-oxide)bis­[di­aqua­dibromido­manganese(II)]–2-methyl­pyridine *N*-oxide (1/2), [Mn_2_Br_4_(C_6_H_7_NO)_2_(H_2_O)_4_]·2C_6_H_7_NO (**II**), bis­(μ-3-methyl­pyridine *N*-oxide)bis­[aqua­dibromido­(3-methyl­pyridine *N*-oxide)manganese(II)], [Mn_2_Br_4_(C_6_H_7_NO)_4_(H_2_O)_2_] (**III**), and bis­(μ-4-methyl­pyridine *N*-oxide)bis­[di­bromido­methanol(4-methyl­pyridine *N*-oxide)manganese(II)], [Mn_2_Br_4_(C_6_H_7_NO)_4_(CH_3_OH)_2_] (**IV**). All the compounds have one unique Mn^II^ atom and form a dimeric complex that contains two Mn^II^ atoms related by a crystallographic inversion center. Pseudo-octa­hedral six-coordinate manganese(II) centers are found in all four compounds. All four compounds form dimers of Mn atoms bridged by the oxygen atom of the PNO ligand. Compounds **I**, **II** and **III** exhibit a bound water of solvation, whereas compound **IV** contains a bound methanol mol­ecule of solvation. Compounds **I**, **III** and **IV** exhibit the same arrangement of mol­ecules around each manganese atom, ligated by two bromide ions, oxygen atoms of two PNO ligands and one solvent mol­ecule, whereas in compound **II** each manganese atom is ligated by two bromide ions, one O atom of a PNO ligand and two water mol­ecules with a second PNO mol­ecule inter­acting with the complex *via* hydrogen bonding through the bound water mol­ecules. All of the compounds form extended hydrogen-bonding networks, and compounds **I**, **II**, and **IV** exhibit offset π-stacking between PNO ligands of neighboring dimers.

## Abstract 2

The synthesis and crystal structures of four dimeric complexes composed of manganese(II) dibromide, a pyridine *N*-oxide and solvent mol­ecules are reported. The pyridine *N-*-oxide, 2-methyl­pyridine *N*-oxide, 3-methyl­pyridine *N*-oxide, and 4-methyl­pyridine *N-*oxide complexes all form similar structures with slight differences owing to the substituent group effects.

## Chemical context   

*N*-oxides have inter­esting binding modes that facilitate the growth of unique coordination structures. Their utility to facilitate organic oxotransfer reactions has been well documented over the years (see, for example, Eppenson, 2003 ▸). Many of these reactions are actually catalyzed by transition-metal inter­actions with the *N*-oxide ligands (see, for example, Moustafa *et al.*, 2014 ▸). Herein, we report four coordination dimers; however, many of these types of structures extend to the formation of coordination polymers. A recent report shows the utility of pyridine *N*-oxide to facilitate coordination polymer formation with both zinc(II) and manganese(II) metal ions with a single bifunctional ligand containing an acetate and *N*-oxide moiety (Ren *et al.*, 2018 ▸). These have been reported by us (Lynch *et al.*, 2018 ▸; Kang *et al.*, 2017 ▸) and others (Sarma *et al.*, 2008 ▸, 2009 ▸; Sarma & Baruah, 2011 ▸).
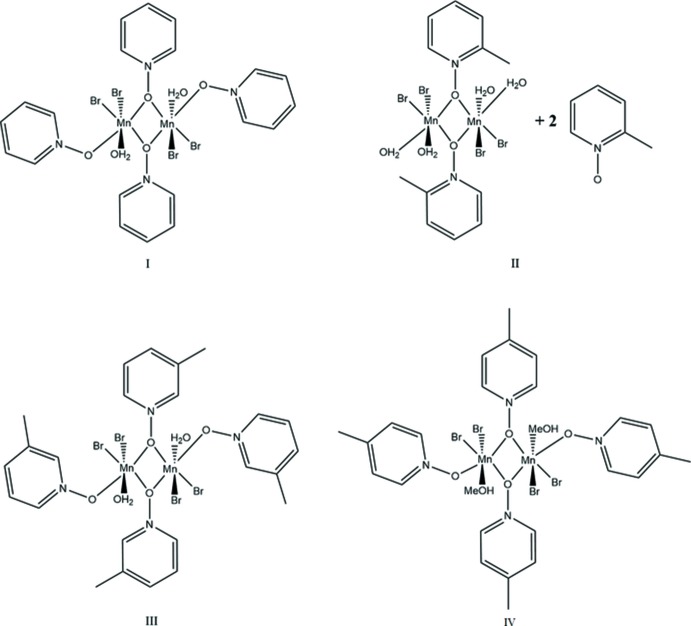


Herein, we report the synthesis and solid-state structures of four pyridine *N*-oxide manganese(II) dimeric complexes, using pyridine *N*-oxide (PNO) and its mono-methyl-substituted forms, 2-methyl­pyridine *N*-oxide (2MePNO), 3-methyl­pyridine *N*-oxide (3MePNO), and 4-methyl­pyridine *N*-oxide (4MePNO). This was done to study the impact of substitution of the pyridine on the two- and three-dimensional solid-state structures, and to compare them to previous structures in which the bromide ions are replaced with chloride ions.

## Structural commentary   

**General structural details**

**Figure 1:**
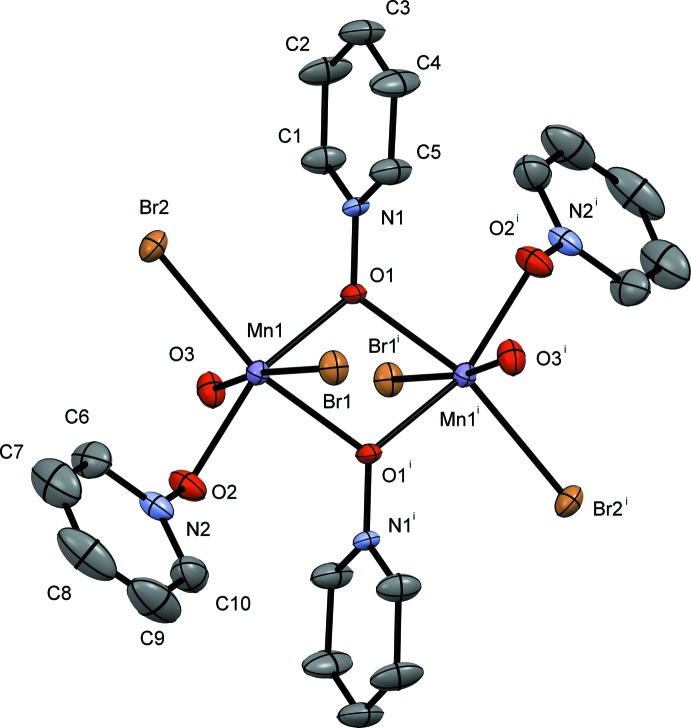
A view of compound **I**, showing the atom labeling. Displacement ellipsoids are drawn at the 50% probability level. H atoms have been omitted for clarity. [Symmetry code: (i) −*x* + 1, −*y* + 1, −*z* + 1]

The pyridine *N*-oxide complexes form dimers consisting of two Mn^II^ atoms related by an inversion center; the dimer contains a six-coordinate metal center at each Mn^II^ ion with four donor oxygen atoms and two bromides. The Mn1⋯Mn1′ dimer is bound *trans* by two μ_2_-1,1-PNO ligands, and the octa­hedral environment is completed by a water mol­ecule of hydration or a solvent mol­ecule, non-bridging PNO ligands, and bromide ions. The dimer is constructed from symmetry-related atoms and mol­ecules using a crystallographic inversion center of the space group (*P* 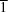 and *P*2_1_/*n*). The mol­ecular structures of compounds **I**, **II**, **III** and **IV** are given in Figs. 1 ▸, 2 ▸, 3 ▸ and 4 ▸, respectively.

**Specific structural details**

Compound **I** (Fig. 1 ▸) crystallizes in the monoclinic space group *P*2_1_/*n*. The Mn—O bond lengths in compound **I** for the bridging PNO ligand are 2.172 (2) and 2.235 (2) Å for Mn1—O1 and Mn1—O1^i^, respectively, which is unremarkable for compounds of Mn^II^ and pyridine *N*-oxide (Sniekers *et al.*, 2017 ▸; Mondal *et al.*, 2012 ▸). The non-bridging Mn1—O2 bond length is 2.099 (3) Å and the bound water Mn1—O3 bond length is 2.312 (3) Å. The bound bromide ions have bond lengths of Mn1—Br1 = 2.7212 (13) Å and Mn1—Br2 = 2.5813 (13) Å; the Mn1—Br1 bond length is significantly longer than Mn1—Br2 as a result of hydrogen-bonding inter­actions that exist with Br1 but not with Br2 (Table 1 ▸). The bridging Mn1 to Mn1^i^ distance is 3.617 (16) Å. The octa­hedral geometry around the Mn atoms is significantly distorted with the O1—Mn1—O1^i^ bond angle measuring 69.66 (9)°; the other bond angles are within *ca* 9° of 90°. These bond angles and bond lengths are similar to those for other Mn^II^ halide PNO structures (Kang *et al.*, 2017 ▸). The dimer also forms an intra­molecular hydrogen bond involving the water O atom, O3, and atom Br1^i^, with a hydrogen bond distance of 2.58 (2) Å [Table 1 ▸; symmetry code: (i) −*x* + 1, −*y* + 1, −*z* + 1].

**Figure 2:**
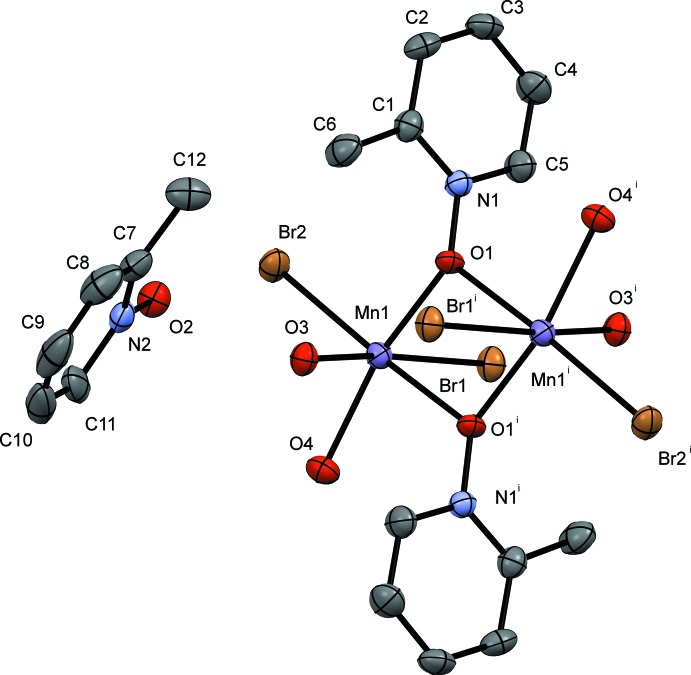
A view of compound **II**, showing the atom labeling. Displacement ellipsoids are drawn at the 50% probability level. H atoms have been omitted for clarity. [Symmetry code: (i) −*x* + 2, −*y* + 1, −*z* + 1]

Compound **II** (Fig. 2 ▸) crystallizes in the triclinic space group *P* 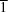. The bond distances observed in compound **II** at Mn1 for the bridging 2MePNO are 2.214 (2) and 2.321 (2) Å for Mn1—O1 and Mn1—O1^i^, respectively. The two bound water mol­ecules have Mn—O bond lengths of 2.237 (3) and 2.157 (3) Å for Mn1—O3 and Mn1—O4, respectively, and are similar to those reported previously (Mondal, *et al.*, 2012 ▸; Lynch, *et al.*, 2018 ▸; Kang *et al.*, 2017 ▸). The bound bromide ions have bond distances of Mn1—Br1 = 2.7009 (7) Å and Mn1—Br2 = 2.6340 (7) Å. In compound **II** both bromide atoms are involved in hydrogen-bonding inter­actions (Table 2 ▸). The Mn1 to Mn1^i^ distance is 3.6128 (11) Å. Once again the octa­hedral geometry around the Mn atoms is significantly distorted with the O1—Mn1—O1^i^ bond angle measuring 74.40 (9)°. The other bond angles are within *ca* 11° of 90°. The dimer forms an intra­molecular hydrogen bond between O3 and Br1^i^ with a hydrogen-bond distance of 2.44 (2) Å [Table 2 ▸; symmetry code: (i) −*x* + 2, −*y* + 1, −*z* + 1]. In the asymmetric unit there is a second PNO mol­ecule inter­acting with the complex *via* hydrogen bonding through the bound water mol­ecules (Table 2 ▸).

**Figure 3:**
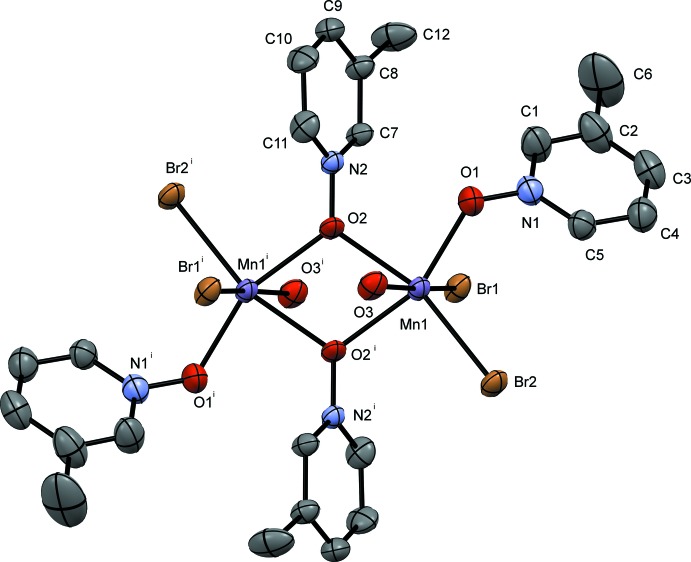
A view of compound **III**, showing the atom labeling. Displacement ellipsoids are drawn at the 50% probability level. H atoms have been omitted for clarity. [Symmetry code: (i) −*x*, −*y* + 1, −*z*]

Compound **III** (Fig. 3 ▸) crystallizes in the triclinic space group *P* 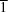 and is very similar to compound **I**. The bond distances observed in compound **III** at Mn1 for the bridging 3MePNO are 2.211 (3) and 2.219 (3) Å for Mn1—O2 and Mn1—O2^i^, respectively. The non-bridging Mn1—O1 bond is 2.129 (3) Å, and the bound water Mn1—O3 bond distance is 2.245 (3) Å. The bound bromide ions have bond distances of Mn1—Br1 = 2.7237 (7) Å and Mn1—Br2 = 2.5687 (7) Å; again the difference in Mn—Br bond distances can be attributed to the hydrogen-bonding inter­actions that exist with Br1 but not with Br2 (Table 3 ▸). The Mn1 to Mn1^i^ distance is 3.6497 (13) Å. The octa­hedral geometry around the Mn atoms is significantly distorted with the O2—Mn1—O2^i^ bond angle measuring 69.05 (11)° the other bond angles are within *ca* 11° of 90°. The dimer forms an intra­molecular hydrogen bond between O3 and Br1^ii^ with a hydrogen-bond distance of 2.55 (2) Å [Table 3 ▸; symmetry code: (ii) −*x*, −*y* + 1, −*z*].

**Figure 4:**
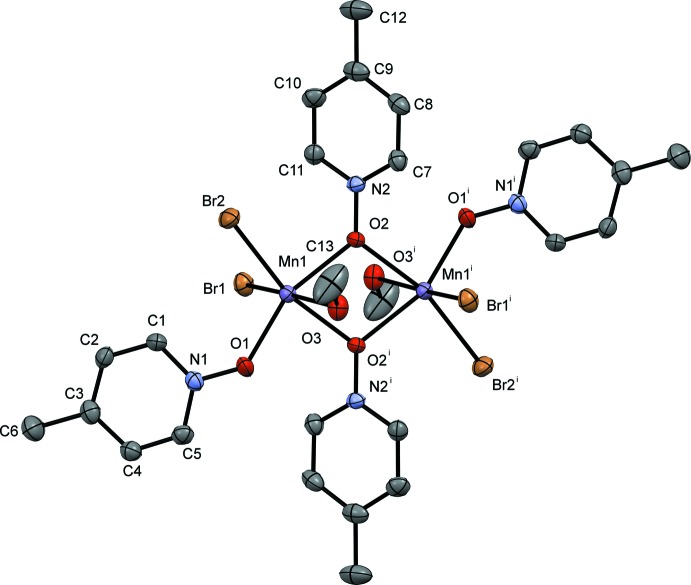
A view of compound **IV**, showing the atom labeling. Displacement ellipsoids are drawn at the 50% probability level. H atoms have been omitted for clarity. [Symmetry code: (i) −*x* + 1, −*y* + 1, −*z* + 1]

Compound **IV** (Fig. 4 ▸) crystallizes in the monoclinic space group *P*2_1_/*n*. The bond distances observed in compound **IV** at Mn1 for the bridging 4MePNO are 2.201 (2) and 2.230 (3) Å for Mn1—O2 and Mn1—O2^i^, respectively. The non-bridging Mn1—O1 bond is 2.116 (3) Å, and the bound methanol Mn1—O3 bond distance is 2.225 (3) Å. The bound bromide ions have bond distances of Mn1—Br1 = 2.7181 (7) Å and Mn1—Br2 2.5806 (7) Å, again the difference in Mn—Br bond distance can be attributed to the hydrogen-bonding inter­actions (Table 4 ▸). The Mn1 to Mn1^i^ distance is 3.61254 (12) Å. The octa­hedral geometry around the Mn atoms is significantly distorted with the O2—Mn1—O2^i^ bond angle measuring 70.77 (11)° the other bond angles are within 13° of 90°. The dimer forms an intra­molecular hydrogen bond between O3 and Br1^i^ with a hydrogen-bond distance of 2.41 (2) Å [Table 4 ▸; symmetry code: (i) −*x* + 1, −*y* + 1, −*z* + 1].

## Supra­molecular features   

**Figure 5:**
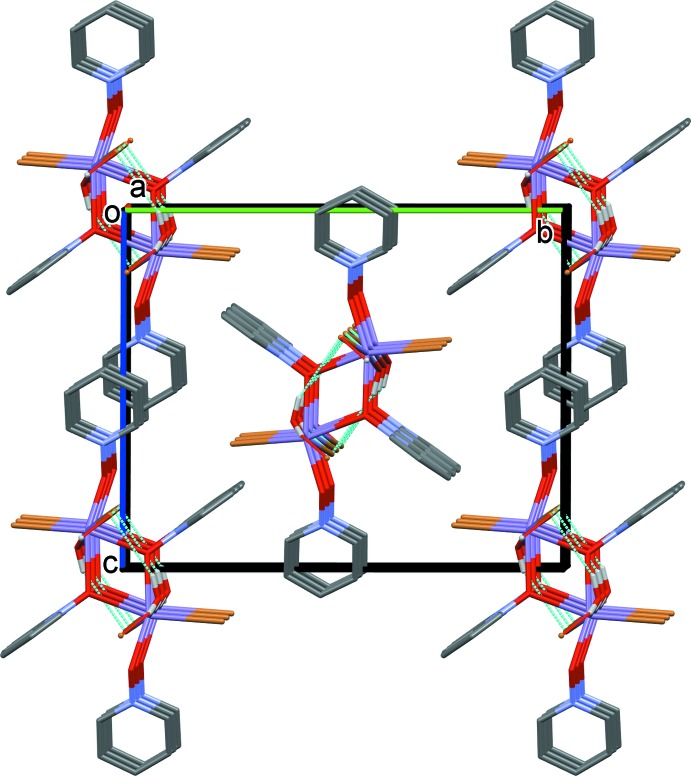
Crystal packing diagram of compound **I**, viewed down the *a* axis. C-bound H atoms have been omitted for clarity. Hydrogen-bonding inter­actions are indicated by dashed lines (Table 1 ▸).

In the crystal of compound **I**, the dimers are linked by O_water_—H⋯Br hydrogen bonds, forming chains parallel to the [100] direction; see Table 1 ▸. The chains are linked by offset π–π inter­actions between inversion-related non-bridging PNO ligands [ring N2/C6–C10; inter-centroid distance = 3.663 (5) Å; offset = 1.399 Å], forming layers parallel to the *ac* plane (Fig. 5 ▸).

**Figure 6:**
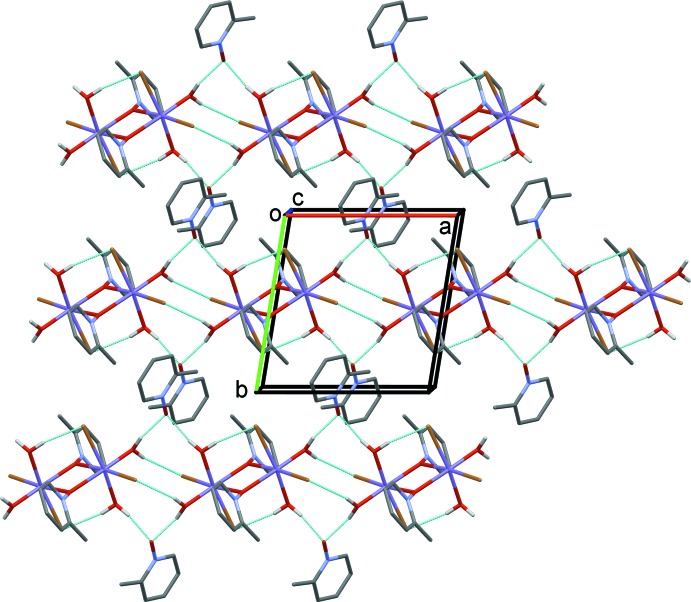
Crystal packing diagram of compound **II**, viewed down the *c* axis. C-bound H atoms have been omitted for clarity. Hydrogen-bonding inter­actions are indicated by dashed lines (Table 2 ▸).

Compound **II** is a dimer with two water mol­ecules bound to each Mn^II^ atom and to only one 2MePNO ligand. The structure has a second 2MePNO mol­ecule not bound to an Mn atom. This unbound 2MePNO is hydrogen-bonded to the bound water mol­ecules of two different dimers, O3⋯O2 = 2.731 (4) Å and O4⋯O2^ii^ = 2.721 (4) Å (Table 2 ▸). Neighboring dimers also form hydrogen bonds between bound water mol­ecules and bromide ions, O3—H3*B*⋯Br1^i^ with a distance of 2.44 (2) Å (Fig. 6 ▸; see Table 2 ▸ for hydrogen-bond details and symmetry codes). Combined, these inter­actions form a hydrogen-bonded chain running parallel to the *a* axis. Neighboring chains are held together through offset π-stacking between the non-bonded 2MePNO ligands (ring N2/C7–C11), with an inter-centroid distance of the stacked aromatic rings of 3.516 (4) Å, so forming layers parallel to the *ac* plane (Fig. 6 ▸).

**Figure 7:**
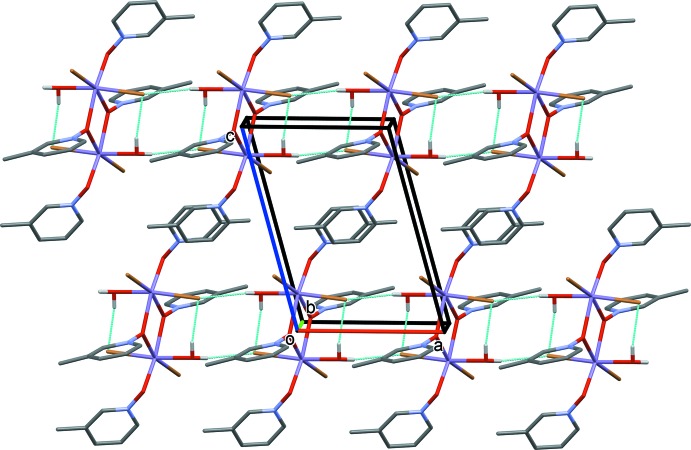
Crystal packing diagram of compound **III**, viewed looking down the *b* axis. C-bound H atoms have been omitted for clarity. Hydrogen-bonding inter­actions are indicated by dashed lines (Table 3 ▸).

The packing in **III** is similar to that for compound **I**; however, the aromatic inter-centroid distance is longer than in the other two compounds, 4.545 (5) Å, with a significant centroid shift of 3.221 (9) Å preventing π-stacking. Neighboring dimers are linked by O—H⋯Br hydrogen-bonds forming chains parallel to the *a* axis. There are two observed inter­actions, O3—H3*A*⋯Br1^i^ with a distance of 2.60 (2) Å and O3—H3*B*⋯Br1^ii^ with a distance of 2.55 (2) Å (Fig. 7 ▸; see Table 3 ▸ for hydrogen-bond details and symmetry codes).

**Figure 8:**
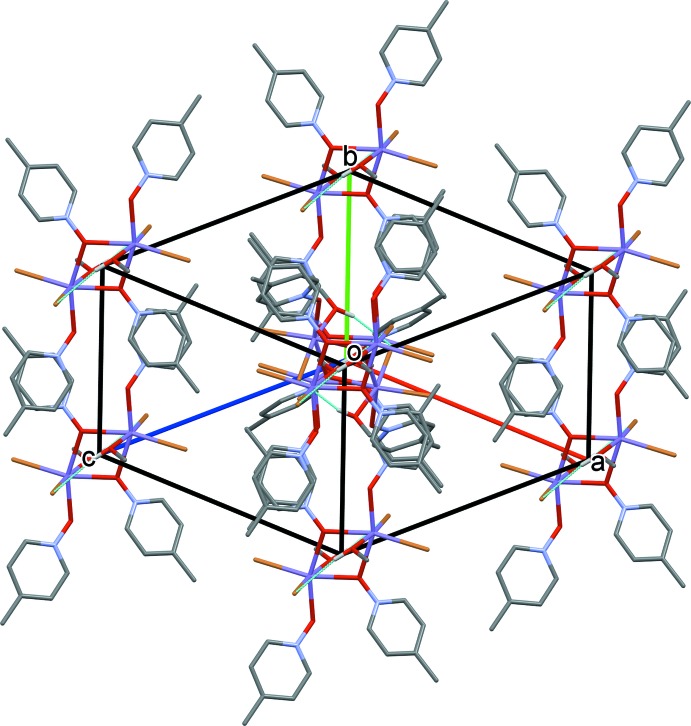
Crystal packing diagram of compound **IV**, viewed along direction [111]. C-bound H atoms have been omitted for clarity. Hydrogen-bonding inter­actions are indicated by dashed lines (Table 4 ▸).

Compound **IV**, a dimeric structure with a bound mol­ecule of methanol replacing the bound water mol­ecule of compound **I** to each of the Mn^II^ atoms, packs very similarly to compound **I** (Fig. 8 ▸ and Table 4 ▸). The inter-centroid distance of the offset π-stacked aromatic rings is 3.824 (5) Å between bridging 4MePNO mol­ecules and non-bridging 4MePNO mol­ecules. This results in the formation of chains running parallel to the *b* axis (Fig. 8 ▸). There is no hydrogen-bonding observed between neighboring dimers in this structure.

## Database survey   

A search in the Cambridge Structural Database (CSD, Version 5.40, November 2018 update; Groom *et al.*, 2016 ▸) for aromatic *N*-oxides and halogen ligands bound to manganese returned six entries (five chlorides and one iodide). Five of these structures contain derivatives of pyridine *N*-oxides and one of them is a 4,4′-dipyridal *N*,*N′*-dioxide (CSD refcode PALYEH; Ghosh *et al.*, 2005 ▸). Three of these structures are the chloride analogs of compounds presented here, *viz*. [MnCl_2_(PNO)(H_2_O)]_*n*_, [MnCl_2_(2MPNO)(H_2_O)]_*n*_, and [MnCl_2_(3MPNO)(H_2_O)_2_]_2_ (VEJLUU, VEJMAB, and VEJMEF, respectively; Kang *et al.*, 2017 ▸), and one is an iodide analog [Mn_2_(PNO)_2_(H_2_O)_6_I_2_]I_2_ (GIWQAF; Shi *et al.*, 2007 ▸). The other two involve functionalized pyridine *N*-oxides; 2-amino (MIRGID; Niu *et al.*, 2001 ▸) and 4-carb­oxy­lic acid (OROZUR; Liu *et al.*, 2010 ▸).

## Synthesis and crystallization   

**Compound I:** Manganese(II) bromide tetra­hydrate (0.320 g, 1.12 mmol) was dissolved in a minimal amount (20 ml) of methanol. Two molar equivalents of pyridine *N*-oxide (PNO; 0.212 g, 2.23 mmol) were also dissolved in methanol. The solutions were mixed and stirred for 10 min and the solvent was allowed to evaporate to produce X-ray quality crystals (yield 0.219 g, 46.4%). Selected IR bands (ATR, FT–IR, KBr composite, cm^−1^) 3470 (*m*, *br*), 1471 (*s*), 1216 (*s*), 833 (*s*) 773 (*m*), 669 (*m*), 558 (*m*). Analysis calculated for C_20_H_24_N_4_Mn_2_Br_4_O_6_: C, 28.40; H, 2.86; N, 6.62%. Found: C, 28.13; H, 2.86; N, 6.50%.

**Compound II:** Manganese(II) bromide tetra­hydrate (0.302 g, 1.05 mmol) was dissolved in a minimal amount (20 ml) of methanol. Two molar equivalents of 2-methyl­pyridine *N*-oxide (2MPNO; 0.230 g, 2.11 mmol) were also dissolved in methanol. The solutions were mixed and stirred for 10 min and the solvent was allowed to evaporate to produce X-ray quality crystals (yield: 0.212 g, 42.9%). Selected IR bands (ATR, FT–IR, KBr composite, cm^−1^) 3349 (*m, br*), 1600 (*m*), 1461 (*s*), 1195 (*s*) 842 (*m*), 772 (*s*), 557 (*m*). Analysis calculated for C_24_H_36_N_4_Mn_2_Br_4_O_8_: C, 30.73; H, 3.87; N, 5.97%. Found: C, 30.30; H, 3.62; N, 6.17%.

**Compound III:** Manganese(II) bromide tetra­hydrate (0.312 g, 1.09 mmol) was dissolved in a minimal amount (20 ml) of methanol. Two molar equivalents of 3-methyl­pyridine *N*-oxide (3MPNO; 0.230 g, 2.12 mmol) were also dissolved in methanol. The solutions were mixed and stirred for 10 min and the solvent was allowed to evaporate to produce a powder (yield: 0.243 g, 49.5%). X-ray quality crystals were grown by recrystallizing a second time by slow evaporation from methanol. Selected IR bands (ATR, FT–IR, KBr composite, cm^−1^) 3373 (*m, br*), 1631 (*s*), 1492 (*m*), 1260 (*m*), 1163(*s*), 943 (*m*), 802 (*m*).

**Compound IV:** Manganese(II) bromide tetra­hydrate (0.302 g; 1.05 mmol) was dissolved in a minimal amount (20 ml) of methanol. Two molar equivalents of 4-methyl­pyridine *N*-oxide (4MPNO; 0.230 g, 2.11 mmol) were also dissolved in methanol. The solutions were mixed and stirred for 10 min and the solvent was allowed to evaporate to produce a powder (yield: 0.215 g, 44.1%). X-ray quality crystals were grown by recrystallizing a second time from methanol with a slower evaporation rate. Selected IR bands (ATR, FT–IR, KBr composite, cm^−1^) 3227 (*m, br*), 3004 (*m*), 1670 (*m*), 1494(*s*), 1213 (*s*), 852(*s*), 763(*s*).

Compounds **I** and **II** have been reported analytically pure, whereas **III** and **IV** were not isolated analytically pure. The FT–IR spectra of the four *N*-oxide complexes all exhibit broad absorbances in the 3500–3100 cm^−1^ region characteristic of the ν(O—H) of the coordinated water or methanol mol­ecules. In addition, the ν(N—O) stretching frequency that is due to the *N*-oxide pyridyl moiety is observed in the region between 1260 and 1195 cm^−1^, as noted previously (Mautner *et al.*, 2017 ▸).

## Refinement   

Crystal data, data collection and structure refinement details are summarized in Table 5 ▸. In order to ensure chemically meaningful O—H distances for the bound water mol­ecules in compounds **I**–**III**, the O—H distances were restrained to 0.84 (2) Å and refined with *U*_iso_(H) = 1.5*U*_eq_(O). In compound **IV**, the hydroxyl H atom was located in a difference-Fourier map and refined with O—H distance restrained to 0.85 (1) Å and with *U*_iso_(H) = 1.5*U*_eq_(O). All carbon-bound H atoms were positioned geometrically and refined as riding: C—H = 0.95–0.98 Å with *U*_iso_(H) = 1.5*U*_eq_(C-meth­yl) and 1.2*U*_eq_(C) for other H atoms.

## Supplementary Material

Crystal structure: contains datablock(s) Global, II, III, IV, I. DOI: 10.1107/S2056989019010557/su5505sup1.cif

Structure factors: contains datablock(s) I. DOI: 10.1107/S2056989019010557/su5505Isup2.hkl

Structure factors: contains datablock(s) II. DOI: 10.1107/S2056989019010557/su5505IIsup3.hkl

Structure factors: contains datablock(s) III. DOI: 10.1107/S2056989019010557/su5505IIIsup4.hkl

Structure factors: contains datablock(s) IV. DOI: 10.1107/S2056989019010557/su5505IVsup5.hkl

CCDC references: 1942967, 1942966, 1942965, 1942964

Additional supporting information:  crystallographic information; 3D view; checkCIF report

## Figures and Tables

**Table 1:** Hydrogen-bond geometry (Å, °) for **I** [Chem scheme1]

*D*—H⋯*A*	*D*—H	H⋯*A*	*D*⋯*A*	*D*—H⋯*A*
O3—H3*A*⋯Br1^i^	0.83 (2)	2.58 (2)	3.372 (3)	159 (4)
O3—H3*B*⋯Br1^ii^	0.84 (4)	2.66 (4)	3.473 (4)	163 (4)

Symmetry codes: (i) 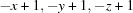; (ii) 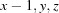.

**Table 2:** Hydrogen-bond geometry (Å, °) for **II** [Chem scheme1]

*D*—H⋯*A*	*D*—H	H⋯*A*	*D*⋯*A*	*D*—H⋯*A*
O3—H3*A*⋯O2	0.85 (2)	1.89 (2)	2.731 (4)	171 (4)
O3—H3*B*⋯Br1^i^	0.86 (2)	2.44 (2)	3.282 (3)	168 (4)
O4—H4*A*⋯O2^ii^	0.85 (2)	1.91 (3)	2.721 (4)	161 (5)
O4—H4*B*⋯Br2^ii^	0.83 (4)	2.59 (4)	3.403 (3)	167 (4)

Symmetry codes: (i) 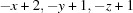; (ii) 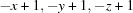.

**Table 3:** Hydrogen-bond geometry (Å, °) for **III** [Chem scheme1]

*D*—H⋯*A*	*D*—H	H⋯*A*	*D*⋯*A*	*D*—H⋯*A*
O3—H3*A*⋯Br1^i^	0.83 (2)	2.60 (2)	3.410 (3)	164 (4)
O3—H3*B*⋯Br1^ii^	0.84 (2)	2.55 (2)	3.386 (3)	172 (5)

Symmetry codes: (i) 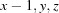; (ii) 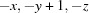.

**Table 4:** Hydrogen-bond geometry (Å, °) for **IV** [Chem scheme1]

*D*—H⋯*A*	*D*—H	H⋯*A*	*D*⋯*A*	*D*—H⋯*A*
O3—H3⋯Br1^i^	0.86 (1)	2.41 (2)	3.255 (3)	166 (3)

Symmetry code: (i) 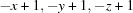.

**Table 5:** Experimental details

	**I**	**II**	**III**	**IV**
Crystal data				
Chemical formula	[Mn_2_Br_4_(C_5_H_5_NO)_4_(H_2_O)_2_]	[Mn_2_Br_4_(C_6_H_7_NO)_2_(H_2_O)_4_]·2C_6_H_7_NO	[Mn_2_Br_4_(C_6_H_7_NO)_4_(H_2_O)_2_]	[Mn_2_Br_4_(C_6_H_7_NO)_4_(CH_4_O)_2_]
*M*_r_	845.95	938.09	902.05	930.11
Crystal system, space group	Monoclinic, *P*2_1_/*n*	Triclinic, *P* 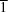	Triclinic, *P* 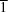	Monoclinic, *P*2_1_/*n*
Temperature (K)	170	170	170	170
*a*, *b*, *c* (Å)	7.736 (4), 15.179 (7), 12.528 (6)	8.9560 (8), 9.7922 (9), 10.2945 (8)	7.6354 (5), 9.9700 (8), 11.898 (1)	13.5384 (7), 9.5354 (4), 13.7292 (7)
α, β, γ (°)	90, 100.055 (4), 90	110.048 (8), 90.336 (7), 98.052 (7)	111.980 (7), 100.360 (6), 97.737 (6)	90, 103.112 (5), 90
*V* (Å^3^)	1448.5 (12)	838.34 (13)	805.71 (12)	1726.15 (15)
*Z*	2	1	1	2
Radiation type	Mo *K*α	Mo *K*α	Mo *K*α	Mo *K*α
μ (mm^−1^)	6.43	5.57	5.79	5.40
Crystal size (mm)	0.5 × 0.5 × 0.2	0.2 × 0.2 × 0.1	0.45 × 0.4 × 0.2	0.4 × 0.4 × 0.4

Data collection				
Diffractometer	Rigaku Mini template	Rigaku XtaLAB mini	Rigaku XtaLAB mini	Rigaku XtaLAB mini
Absorption correction	Multi-scan (*REQAB*; Rigaku, 1998 ▸)	Multi-scan (*CrysAlis PRO*; Rigaku Oxford Diffraction, 2018 ▸)	Multi-scan (*CrysAlis PRO*; Rigaku Oxford Diffraction, 2018 ▸)	Multi-scan (*CrysAlis PRO*; Rigaku Oxford Diffraction, 2018 ▸)
*T*_min_, *T*_max_	0.066, 0.114	0.580, 1.000	0.319, 1.000	0.659, 1.000
No. of measured, independent and observed [*I* > 2σ(*I*)] reflections	15117, 3305, 3024	8918, 3838, 2931	8460, 3672, 2875	17754, 3964, 3175
*R*_int_	0.178	0.053	0.036	0.061
(sin θ/λ)_max_ (Å^−1^)	0.650	0.649	0.649	0.649

Refinement				
*R*[*F* ^2^ > 2σ(*F* ^2^)], *wR*(*F* ^2^), *S*	0.043, 0.110, 1.13	0.037, 0.080, 1.00	0.040, 0.098, 1.02	0.043, 0.112, 1.03
No. of reflections	3305	3838	3672	3964
No. of parameters	172	208	191	196
No. of restraints	2	4	2	3
H-atom treatment	H atoms treated by a mixture of independent and constrained refinement	H atoms treated by a mixture of independent and constrained refinement	H atoms treated by a mixture of independent and constrained refinement	H atoms treated by a mixture of independent and constrained refinement
Δρ_max_, Δρ_min_ (e Å^−3^)	1.43, −1.15	0.72, −0.62	1.66, −0.84	1.64, −0.74

Computer programs: *CrysAlis PRO* (Rigaku Oxford Diffraction, 2018 ▸), *SHELXT* (Sheldrick, 2015*a* ▸), *SHELXL* (Sheldrick, 2015*b* ▸), *OLEX2* (Dolomanov *et al.*, 2009 ▸) and *Mercury* (Macrae *et al.*, 2008 ▸).

## supplementary crystallographic information

### Bis(µ-pyridine N-oxide)bis[aquadibromido(pyridine N-oxide)manganese(II)] (I) Crystal data

**Table:**

[Mn_2_Br_4_(C_5_H_5_NO)_4_(H_2_O)_2_]	*F*(000) = 820
*M**_r_* = 845.95	*D*_x_ = 1.940 Mg m^−^^3^
Monoclinic, *P*2_1_/*n*	Mo *K*α radiation, λ = 0.71073 Å
*a* = 7.736 (4) Å	Cell parameters from 4250 reflections
*b* = 15.179 (7) Å	θ = 2.1–27.5°
*c* = 12.528 (6) Å	µ = 6.43 mm^−^^1^
β = 100.055 (4)°	*T* = 170 K
*V* = 1448.5 (12) Å^3^	Prism, colorless
*Z* = 2	0.5 × 0.5 × 0.2 mm

### Bis(µ-pyridine N-oxide)bis[aquadibromido(pyridine N-oxide)manganese(II)] (I) Data collection

**Table:**

Rigaku Mini template diffractometer	3305 independent reflections
Radiation source: Sealed Tube	3024 reflections with *I* > 2σ(*I*)
Graphite Monochromator monochromator	*R*_int_ = 0.178
Detector resolution: 13.6612 pixels mm^-1^	θ_max_ = 27.5°, θ_min_ = 2.1°
profile data from ω–scans	*h* = −10→10
Absorption correction: multi-scan (*REQAB*; Rigaku, 1998)	*k* = −19→19
*T*_min_ = 0.066, *T*_max_ = 0.114	*l* = −16→16
15117 measured reflections	

### Bis(µ-pyridine N-oxide)bis[aquadibromido(pyridine N-oxide)manganese(II)] (I) Refinement

**Table:**

Refinement on *F*^2^	Hydrogen site location: mixed
Least-squares matrix: full	H atoms treated by a mixture of independent and constrained refinement
*R*[*F*^2^ > 2σ(*F*^2^)] = 0.043	*w* = 1/[σ^2^(*F*_o_^2^) + (0.0191*P*)^2^ + 0.4786*P*] where *P* = (*F*_o_^2^ + 2*F*_c_^2^)/3
*wR*(*F*^2^) = 0.110	(Δ/σ)_max_ = 0.001
*S* = 1.13	Δρ_max_ = 1.43 e Å^−^^3^
3305 reflections	Δρ_min_ = −1.15 e Å^−^^3^
172 parameters	Extinction correction: (SHELXL-2018/1; Sheldrick, 2015b), Fc^*^=kFc[1+0.001xFc^2^λ^3^/sin(2θ)]^-1/4^
2 restraints	Extinction coefficient: 0.0140 (11)
Primary atom site location: dual	

### Bis(µ-pyridine N-oxide)bis[aquadibromido(pyridine N-oxide)manganese(II)] (I) Special details

**Table:**

Geometry. All esds (except the esd in the dihedral angle between two l.s. planes) are estimated using the full covariance matrix. The cell esds are taken into account individually in the estimation of esds in distances, angles and torsion angles; correlations between esds in cell parameters are only used when they are defined by crystal symmetry. An approximate (isotropic) treatment of cell esds is used for estimating esds involving l.s. planes.

### Bis(µ-pyridine N-oxide)bis[aquadibromido(pyridine N-oxide)manganese(II)] (I) Fractional atomic coordinates and isotropic or equivalent isotropic displacement parameters (Å^2^)

**Table:**

	*x*	*y*	*z*	*U*_iso_*/*U*_eq_	
Br1	0.88172 (4)	0.48605 (2)	0.66632 (3)	0.02264 (14)	
C1	0.4849 (5)	0.3093 (3)	0.3370 (3)	0.0303 (8)	
H1	0.363028	0.310997	0.339833	0.036*	
Mn1	0.54254 (7)	0.43050 (3)	0.61896 (4)	0.01589 (16)	
O1	0.5247 (3)	0.43379 (14)	0.44408 (17)	0.0171 (5)	
N1	0.5922 (4)	0.36998 (16)	0.3881 (2)	0.0160 (5)	
Br2	0.61302 (5)	0.26431 (2)	0.63521 (3)	0.02396 (15)	
N2	0.5951 (4)	0.4668 (2)	0.8619 (2)	0.0250 (6)	
O2	0.4813 (4)	0.46894 (19)	0.7689 (2)	0.0296 (6)	
C2	0.5520 (6)	0.2438 (3)	0.2796 (4)	0.0403 (10)	
H2	0.476706	0.199462	0.243743	0.048*	
C3	0.7280 (6)	0.2428 (3)	0.2744 (4)	0.0377 (10)	
H3	0.774934	0.198354	0.234356	0.045*	
O3	0.2446 (4)	0.40522 (18)	0.5691 (2)	0.0260 (6)	
C4	0.8354 (6)	0.3068 (3)	0.3280 (4)	0.0440 (12)	
H4	0.957332	0.307435	0.325343	0.053*	
C5	0.7628 (5)	0.3702 (3)	0.3855 (4)	0.0339 (9)	
H5	0.835724	0.414384	0.423704	0.041*	
C6	0.6494 (6)	0.3886 (3)	0.9054 (3)	0.0385 (10)	
H6	0.610967	0.335631	0.868308	0.046*	
C7	0.7611 (8)	0.3855 (4)	1.0040 (4)	0.0570 (14)	
H7	0.800377	0.330116	1.034421	0.068*	
C8	0.8162 (7)	0.4620 (5)	1.0588 (4)	0.0627 (17)	
H8	0.892059	0.460197	1.127146	0.075*	
C9	0.7581 (8)	0.5413 (4)	1.0116 (4)	0.0600 (16)	
H9	0.792711	0.594992	1.048066	0.072*	
C10	0.6498 (7)	0.5426 (3)	0.9116 (4)	0.0396 (10)	
H10	0.613977	0.597312	0.877878	0.048*	
H3A	0.214 (6)	0.418 (3)	0.5042 (18)	0.037 (13)*	
H3B	0.172 (5)	0.424 (3)	0.606 (4)	0.042 (14)*	

### Bis(µ-pyridine N-oxide)bis[aquadibromido(pyridine N-oxide)manganese(II)] (I) Atomic displacement parameters (Å^2^)

**Table:**

	*U*^11^	*U*^22^	*U*^33^	*U*^12^	*U*^13^	*U*^23^
Br1	0.0170 (2)	0.0266 (2)	0.0235 (2)	−0.00262 (13)	0.00117 (15)	0.00210 (12)
C1	0.0210 (18)	0.030 (2)	0.038 (2)	−0.0009 (15)	−0.0002 (16)	−0.0156 (16)
Mn1	0.0175 (3)	0.0151 (3)	0.0155 (3)	0.00165 (18)	0.0039 (2)	0.00189 (17)
O1	0.0237 (12)	0.0126 (10)	0.0161 (10)	0.0056 (9)	0.0062 (9)	−0.0010 (8)
N1	0.0210 (14)	0.0101 (12)	0.0172 (12)	0.0016 (11)	0.0046 (11)	−0.0037 (10)
Br2	0.0256 (2)	0.0155 (2)	0.0302 (2)	0.00169 (13)	0.00347 (16)	0.00366 (12)
N2	0.0240 (16)	0.0354 (17)	0.0165 (13)	0.0059 (13)	0.0064 (12)	−0.0019 (12)
O2	0.0298 (15)	0.0420 (16)	0.0175 (11)	0.0119 (12)	0.0050 (11)	0.0009 (11)
C2	0.032 (2)	0.031 (2)	0.054 (3)	0.0001 (18)	−0.0029 (19)	−0.025 (2)
C3	0.038 (2)	0.029 (2)	0.047 (2)	0.0010 (18)	0.012 (2)	−0.0200 (18)
O3	0.0220 (13)	0.0305 (14)	0.0263 (13)	0.0033 (11)	0.0063 (11)	0.0089 (11)
C4	0.026 (2)	0.035 (2)	0.077 (3)	−0.0021 (18)	0.025 (2)	−0.023 (2)
C5	0.026 (2)	0.0254 (19)	0.053 (2)	−0.0056 (16)	0.0161 (19)	−0.0176 (17)
C6	0.044 (3)	0.039 (2)	0.030 (2)	0.016 (2)	0.0008 (18)	0.0047 (17)
C7	0.053 (3)	0.084 (4)	0.032 (2)	0.029 (3)	−0.001 (2)	0.012 (2)
C8	0.032 (3)	0.127 (5)	0.027 (2)	0.003 (3)	−0.001 (2)	−0.013 (3)
C9	0.055 (3)	0.085 (4)	0.043 (3)	−0.030 (3)	0.016 (3)	−0.031 (3)
C10	0.047 (3)	0.038 (2)	0.036 (2)	−0.011 (2)	0.014 (2)	−0.0076 (18)

### Bis(µ-pyridine N-oxide)bis[aquadibromido(pyridine N-oxide)manganese(II)] (I) Geometric parameters (Å, º)

**Table:**

Mn1—Br1	2.7212 (13)	C3—H3	0.9500
C1—H1	0.9500	C3—C4	1.375 (6)
C1—N1	1.327 (5)	O3—H3A	0.829 (19)
C1—C2	1.381 (5)	O3—H3B	0.829 (19)
Mn1—O1	2.172 (2)	C4—H4	0.9500
Mn1—O1^i^	2.235 (2)	C4—C5	1.379 (5)
Mn1—Br2	2.5813 (13)	C5—H5	0.9500
Mn1—O2	2.099 (3)	C6—H6	0.9500
Mn1—O3	2.312 (3)	C6—C7	1.380 (7)
O1—N1	1.353 (3)	C7—H7	0.9500
N1—C5	1.326 (5)	C7—C8	1.378 (8)
N2—O2	1.332 (4)	C8—H8	0.9500
N2—C6	1.342 (5)	C8—C9	1.381 (9)
N2—C10	1.342 (5)	C9—H9	0.9500
C2—H2	0.9500	C9—C10	1.381 (8)
C2—C3	1.374 (6)	C10—H10	0.9500
			
N1—C1—H1	120.3	C3—C2—C1	119.9 (4)
N1—C1—C2	119.4 (4)	C3—C2—H2	120.1
C2—C1—H1	120.3	C2—C3—H3	120.4
O1—Mn1—Br1	95.84 (7)	C2—C3—C4	119.2 (3)
O1^i^—Mn1—Br1	87.00 (7)	C4—C3—H3	120.4
O1—Mn1—O1^i^	69.66 (9)	Mn1—O3—H3A	109 (3)
O1—Mn1—Br2	94.44 (6)	Mn1—O3—H3B	122 (4)
O1^i^—Mn1—Br2	164.07 (6)	H3A—O3—H3B	111 (5)
O1^i^—Mn1—O3	84.14 (10)	C3—C4—H4	120.6
O1—Mn1—O3	81.19 (9)	C3—C4—C5	118.8 (4)
Br2—Mn1—Br1	95.93 (3)	C5—C4—H4	120.6
O2—Mn1—Br1	94.50 (9)	N1—C5—C4	120.6 (4)
O2—Mn1—O1^i^	89.15 (10)	N1—C5—H5	119.7
O2—Mn1—O1	155.81 (10)	C4—C5—H5	119.7
O2—Mn1—Br2	106.17 (8)	N2—C6—H6	120.1
O2—Mn1—O3	85.28 (11)	N2—C6—C7	119.8 (5)
O3—Mn1—Br1	171.14 (7)	C7—C6—H6	120.1
O3—Mn1—Br2	92.63 (7)	C6—C7—H7	119.7
Mn1—O1—Mn1^i^	110.34 (9)	C8—C7—C6	120.5 (5)
N1—O1—Mn1	122.99 (18)	C8—C7—H7	119.7
N1—O1—Mn1^i^	124.32 (17)	C7—C8—H8	120.9
C1—N1—O1	118.8 (3)	C7—C8—C9	118.2 (5)
C5—N1—C1	122.1 (3)	C9—C8—H8	120.9
C5—N1—O1	119.1 (3)	C8—C9—H9	119.9
O2—N2—C6	119.2 (3)	C10—C9—C8	120.1 (5)
O2—N2—C10	119.4 (4)	C10—C9—H9	119.9
C6—N2—C10	121.3 (4)	N2—C10—C9	120.0 (5)
N2—O2—Mn1	123.9 (2)	N2—C10—H10	120.0
C1—C2—H2	120.1	C9—C10—H10	120.0

Symmetry code: (i) −*x*+1, −*y*+1, −*z*+1.

### Bis(µ-pyridine N-oxide)bis[aquadibromido(pyridine N-oxide)manganese(II)] (I) Hydrogen-bond geometry (Å, º)

**Table:**

*D*—H···*A*	*D*—H	H···*A*	*D*···*A*	*D*—H···*A*
O3—H3*A*···Br1^i^	0.83 (2)	2.58 (2)	3.372 (3)	159 (4)
O3—H3*B*···Br1^ii^	0.84 (4)	2.66 (4)	3.473 (4)	163 (4)

Symmetry codes: (i) −*x*+1, −*y*+1, −*z*+1; (ii) *x*−1, *y*, *z*.

### Bis(µ-2-methylpyridine N-oxide)bis[diaquadibromidomanganese(II)]–2-methylpyridine N-oxide (1/2) (II) Crystal data

**Table:**

[Mn_2_Br_4_(C_6_H_7_NO)_2_(H_2_O)_4_]·2C_6_H_7_NO	*Z* = 1
*M**_r_* = 938.09	*F*(000) = 462
Triclinic, *P*1	*D*_x_ = 1.858 Mg m^−^^3^
*a* = 8.9560 (8) Å	Mo *K*α radiation, λ = 0.71073 Å
*b* = 9.7922 (9) Å	Cell parameters from 4291 reflections
*c* = 10.2945 (8) Å	θ = 2.3–33.2°
α = 110.048 (8)°	µ = 5.57 mm^−^^1^
β = 90.336 (7)°	*T* = 170 K
γ = 98.052 (7)°	Block, clear light yellow
*V* = 838.34 (13) Å^3^	0.2 × 0.2 × 0.1 mm

### Bis(µ-2-methylpyridine N-oxide)bis[diaquadibromidomanganese(II)]–2-methylpyridine N-oxide (1/2) (II) Data collection

**Table:**

Rigaku XtaLAB mini diffractometer	3838 independent reflections
Radiation source: fine-focus sealed X-ray tube, Enhance (Mo) X-ray Source	2931 reflections with *I* > 2σ(*I*)
Graphite Monochromator monochromator	*R*_int_ = 0.053
Detector resolution: 13.6612 pixels mm^-1^	θ_max_ = 27.5°, θ_min_ = 2.1°
profile data from ω–scans	*h* = −11→11
Absorption correction: multi-scan (CrysAlisPro; Rigaku Oxford Diffraction, 2018)	*k* = −12→12
*T*_min_ = 0.580, *T*_max_ = 1.000	*l* = −13→13
8918 measured reflections	

### Bis(µ-2-methylpyridine N-oxide)bis[diaquadibromidomanganese(II)]–2-methylpyridine N-oxide (1/2) (II) Refinement

**Table:**

Refinement on *F*^2^	Primary atom site location: dual
Least-squares matrix: full	Hydrogen site location: mixed
*R*[*F*^2^ > 2σ(*F*^2^)] = 0.037	H atoms treated by a mixture of independent and constrained refinement
*wR*(*F*^2^) = 0.080	*w* = 1/[σ^2^(*F*_o_^2^) + (0.0319*P*)^2^] where *P* = (*F*_o_^2^ + 2*F*_c_^2^)/3
*S* = 1.00	(Δ/σ)_max_ = 0.001
3838 reflections	Δρ_max_ = 0.72 e Å^−^^3^
208 parameters	Δρ_min_ = −0.61 e Å^−^^3^
4 restraints	

### Bis(µ-2-methylpyridine N-oxide)bis[diaquadibromidomanganese(II)]–2-methylpyridine N-oxide (1/2) (II) Special details

**Table:**

Geometry. All esds (except the esd in the dihedral angle between two l.s. planes) are estimated using the full covariance matrix. The cell esds are taken into account individually in the estimation of esds in distances, angles and torsion angles; correlations between esds in cell parameters are only used when they are defined by crystal symmetry. An approximate (isotropic) treatment of cell esds is used for estimating esds involving l.s. planes.

### Bis(µ-2-methylpyridine N-oxide)bis[diaquadibromidomanganese(II)]–2-methylpyridine N-oxide (1/2) (II) Fractional atomic coordinates and isotropic or equivalent isotropic displacement parameters (Å^2^)

**Table:**

	*x*	*y*	*z*	*U*_iso_*/*U*_eq_	
Br1	0.97900 (4)	0.80035 (4)	0.74971 (4)	0.02737 (11)	
O1	1.0009 (3)	0.4219 (3)	0.5880 (2)	0.0216 (5)	
C1	0.9673 (4)	0.2520 (4)	0.7046 (4)	0.0271 (8)	
Mn1	0.82268 (6)	0.55061 (6)	0.56887 (5)	0.02123 (13)	
N1	1.0302 (3)	0.3845 (3)	0.6999 (3)	0.0219 (6)	
Br2	0.62884 (4)	0.49697 (4)	0.74319 (4)	0.03065 (11)	
C2	1.0052 (4)	0.2179 (4)	0.8211 (4)	0.0304 (9)	
H2	0.962787	0.125920	0.827065	0.037*	
O3	0.7281 (3)	0.3472 (3)	0.3927 (3)	0.0267 (6)	
H3A	0.647 (3)	0.288 (4)	0.388 (4)	0.043 (13)*	
H3B	0.799 (3)	0.297 (4)	0.359 (4)	0.036 (12)*	
C3	1.1028 (5)	0.3150 (4)	0.9273 (4)	0.0329 (9)	
H3	1.127202	0.290351	1.005596	0.039*	
C4	1.1643 (5)	0.4487 (5)	0.9180 (4)	0.0368 (10)	
H4	1.231315	0.517477	0.990012	0.044*	
O4	0.6790 (3)	0.6595 (3)	0.4807 (3)	0.0268 (6)	
H4A	0.642 (6)	0.726 (4)	0.542 (4)	0.09 (2)*	
H4B	0.614 (5)	0.614 (6)	0.417 (4)	0.10 (2)*	
C5	1.1269 (4)	0.4809 (4)	0.8023 (4)	0.0320 (9)	
H5	1.169778	0.571996	0.794612	0.038*	
C6	0.8633 (5)	0.1536 (4)	0.5885 (4)	0.0377 (10)	
H6A	0.769395	0.194740	0.589890	0.057*	
H6B	0.840725	0.056545	0.597618	0.057*	
H6C	0.910470	0.144060	0.500794	0.057*	
N2	0.4507 (3)	0.0303 (3)	0.2438 (3)	0.0262 (7)	
O2	0.4865 (3)	0.1344 (3)	0.3682 (3)	0.0288 (6)	
C7	0.5133 (4)	−0.0960 (4)	0.2104 (4)	0.0292 (9)	
C8	0.4748 (6)	−0.2028 (5)	0.0806 (4)	0.0455 (12)	
H8	0.518213	−0.290783	0.054919	0.055*	
C9	0.3744 (6)	−0.1829 (6)	−0.0117 (5)	0.0544 (14)	
H9	0.349499	−0.256046	−0.100515	0.065*	
C10	0.3105 (5)	−0.0539 (6)	0.0278 (5)	0.0516 (13)	
H10	0.238985	−0.039227	−0.032830	0.062*	
C11	0.3517 (4)	0.0509 (5)	0.1542 (4)	0.0378 (10)	
H11	0.310341	0.140125	0.180201	0.045*	
C12	0.6228 (5)	−0.1073 (4)	0.3143 (4)	0.0383 (10)	
H12A	0.713697	−0.035107	0.324504	0.057*	
H12B	0.650204	−0.206492	0.283156	0.057*	
H12C	0.576598	−0.087782	0.403703	0.057*	

### Bis(µ-2-methylpyridine N-oxide)bis[diaquadibromidomanganese(II)]–2-methylpyridine N-oxide (1/2) (II) Atomic displacement parameters (Å^2^)

**Table:**

	*U*^11^	*U*^22^	*U*^33^	*U*^12^	*U*^13^	*U*^23^
Br1	0.0266 (2)	0.0235 (2)	0.0261 (2)	0.00076 (15)	0.00103 (15)	0.00222 (15)
O1	0.0243 (13)	0.0249 (13)	0.0179 (12)	0.0027 (11)	−0.0006 (10)	0.0110 (10)
C1	0.0272 (19)	0.024 (2)	0.028 (2)	0.0024 (16)	0.0022 (16)	0.0072 (16)
Mn1	0.0213 (3)	0.0217 (3)	0.0198 (3)	0.0016 (2)	−0.0009 (2)	0.0067 (2)
N1	0.0236 (15)	0.0223 (16)	0.0203 (15)	0.0014 (13)	0.0008 (13)	0.0088 (13)
Br2	0.0294 (2)	0.0379 (2)	0.0225 (2)	−0.00298 (17)	0.00138 (16)	0.01083 (17)
C2	0.040 (2)	0.026 (2)	0.031 (2)	0.0067 (18)	0.0077 (18)	0.0170 (18)
O3	0.0247 (14)	0.0246 (14)	0.0269 (14)	−0.0001 (12)	0.0010 (12)	0.0055 (12)
C3	0.040 (2)	0.039 (2)	0.027 (2)	0.0095 (19)	0.0009 (18)	0.0198 (19)
C4	0.041 (2)	0.041 (2)	0.025 (2)	−0.002 (2)	−0.0076 (18)	0.0103 (19)
O4	0.0305 (15)	0.0269 (15)	0.0228 (15)	0.0069 (13)	−0.0031 (13)	0.0074 (12)
C5	0.035 (2)	0.028 (2)	0.030 (2)	−0.0046 (18)	−0.0073 (18)	0.0099 (17)
C6	0.046 (3)	0.029 (2)	0.035 (2)	−0.0069 (19)	−0.003 (2)	0.0126 (19)
N2	0.0229 (16)	0.0293 (18)	0.0237 (16)	−0.0054 (14)	0.0025 (13)	0.0096 (14)
O2	0.0316 (14)	0.0243 (14)	0.0255 (14)	0.0036 (12)	0.0051 (12)	0.0024 (11)
C7	0.038 (2)	0.024 (2)	0.0230 (19)	−0.0034 (17)	0.0049 (17)	0.0080 (16)
C8	0.068 (3)	0.028 (2)	0.030 (2)	−0.012 (2)	0.005 (2)	0.0046 (19)
C9	0.062 (3)	0.057 (3)	0.027 (2)	−0.036 (3)	−0.005 (2)	0.009 (2)
C10	0.032 (2)	0.088 (4)	0.036 (3)	−0.017 (3)	−0.008 (2)	0.033 (3)
C11	0.0185 (19)	0.059 (3)	0.042 (3)	0.0064 (19)	0.0035 (18)	0.024 (2)
C12	0.054 (3)	0.035 (2)	0.030 (2)	0.018 (2)	0.007 (2)	0.0113 (19)

### Bis(µ-2-methylpyridine N-oxide)bis[diaquadibromidomanganese(II)]–2-methylpyridine N-oxide (1/2) (II) Geometric parameters (Å, º)

**Table:**

Mn1—Br1	2.7009 (7)	O4—H4B	0.83 (2)
Mn1—O1	2.214 (2)	C5—H5	0.9500
O1—Mn1^i^	2.321 (2)	C6—H6A	0.9800
O1—N1	1.358 (3)	C6—H6B	0.9800
C1—N1	1.357 (4)	C6—H6C	0.9800
C1—C2	1.402 (5)	N2—O2	1.338 (4)
C1—C6	1.473 (5)	N2—C7	1.366 (5)
Mn1—Br2	2.6340 (7)	N2—C11	1.359 (5)
Mn1—O3	2.237 (3)	C7—C8	1.390 (5)
Mn1—O4	2.157 (3)	C7—C12	1.490 (5)
N1—C5	1.353 (5)	C8—H8	0.9500
C2—H2	0.9500	C8—C9	1.384 (7)
C2—C3	1.381 (5)	C9—H9	0.9500
O3—H3A	0.852 (19)	C9—C10	1.394 (7)
O3—H3B	0.857 (19)	C10—H10	0.9500
C3—H3	0.9500	C10—C11	1.362 (6)
C3—C4	1.383 (5)	C11—H11	0.9500
C4—H4	0.9500	C12—H12A	0.9800
C4—C5	1.382 (5)	C12—H12B	0.9800
O4—H4A	0.846 (19)	C12—H12C	0.9800
			
O1—Mn1—O1^i^	74.40 (9)	Mn1—O4—H4A	112 (4)
Mn1—O1—Mn1^i^	105.60 (9)	Mn1—O4—H4B	123 (4)
N1—O1—Mn1	125.53 (19)	H4A—O4—H4B	110 (5)
N1—O1—Mn1^i^	124.62 (18)	N1—C5—C4	121.0 (3)
N1—C1—C2	117.5 (3)	N1—C5—H5	119.5
N1—C1—C6	118.5 (3)	C4—C5—H5	119.5
C2—C1—C6	124.1 (3)	C1—C6—H6A	109.5
O1—Mn1—Br1	91.54 (6)	C1—C6—H6B	109.5
O1^i^—Mn1—Br1	85.90 (6)	C1—C6—H6C	109.5
O1—Mn1—Br2	101.36 (6)	H6A—C6—H6B	109.5
O1^i^—Mn1—Br2	175.09 (6)	H6A—C6—H6C	109.5
O1—Mn1—O3	84.60 (9)	H6B—C6—H6C	109.5
Br2—Mn1—Br1	96.79 (2)	O2—N2—C7	119.0 (3)
O3—Mn1—Br1	169.21 (8)	O2—N2—C11	119.7 (3)
O3—Mn1—O1^i^	83.36 (9)	C11—N2—C7	121.3 (3)
O3—Mn1—Br2	93.85 (7)	N2—C7—C8	118.2 (4)
O4—Mn1—Br1	95.42 (8)	N2—C7—C12	117.4 (3)
O4—Mn1—O1^i^	87.70 (9)	C8—C7—C12	124.4 (4)
O4—Mn1—O1	160.29 (10)	C7—C8—H8	119.5
O4—Mn1—Br2	96.11 (8)	C9—C8—C7	121.1 (5)
O4—Mn1—O3	85.19 (10)	C9—C8—H8	119.5
C1—N1—O1	120.1 (3)	C8—C9—H9	120.5
C5—N1—O1	117.8 (3)	C8—C9—C10	118.9 (4)
C5—N1—C1	122.0 (3)	C10—C9—H9	120.5
C1—C2—H2	119.2	C9—C10—H10	120.4
C3—C2—C1	121.5 (3)	C11—C10—C9	119.3 (4)
C3—C2—H2	119.2	C11—C10—H10	120.4
Mn1—O3—H3A	128 (3)	N2—C11—C10	121.2 (4)
Mn1—O3—H3B	110 (3)	N2—C11—H11	119.4
H3A—O3—H3B	109 (4)	C10—C11—H11	119.4
C2—C3—H3	120.5	C7—C12—H12A	109.5
C2—C3—C4	119.0 (3)	C7—C12—H12B	109.5
C4—C3—H3	120.5	C7—C12—H12C	109.5
C3—C4—H4	120.5	H12A—C12—H12B	109.5
C5—C4—C3	119.0 (4)	H12A—C12—H12C	109.5
C5—C4—H4	120.5	H12B—C12—H12C	109.5

Symmetry code: (i) −*x*+2, −*y*+1, −*z*+1.

### Bis(µ-2-methylpyridine N-oxide)bis[diaquadibromidomanganese(II)]–2-methylpyridine N-oxide (1/2) (II) Hydrogen-bond geometry (Å, º)

**Table:**

*D*—H···*A*	*D*—H	H···*A*	*D*···*A*	*D*—H···*A*
O3—H3*A*···O2	0.85 (2)	1.89 (2)	2.731 (4)	171 (4)
O3—H3*B*···Br1^i^	0.86 (2)	2.44 (2)	3.282 (3)	168 (4)
O4—H4*A*···O2^ii^	0.85 (2)	1.91 (3)	2.721 (4)	161 (5)
O4—H4*B*···Br2^ii^	0.83 (4)	2.59 (4)	3.403 (3)	167 (4)

Symmetry codes: (i) −*x*+2, −*y*+1, −*z*+1; (ii) −*x*+1, −*y*+1, −*z*+1.

### Bis(µ-3-methylpyridine N-oxide)bis[aquadibromido(3-methylpyridine N-oxide)manganese(II)] (III) Crystal data

**Table:**

[Mn_2_Br_4_(C_6_H_7_NO)_4_(H_2_O)_2_]	*Z* = 1
*M**_r_* = 902.05	*F*(000) = 442
Triclinic, *P*1	*D*_x_ = 1.859 Mg m^−^^3^
*a* = 7.6354 (5) Å	Mo *K*α radiation, λ = 0.71073 Å
*b* = 9.9700 (8) Å	Cell parameters from 4991 reflections
*c* = 11.898 (1) Å	θ = 2.2–33.1°
α = 111.980 (7)°	µ = 5.79 mm^−^^1^
β = 100.360 (6)°	*T* = 170 K
γ = 97.737 (6)°	Plate, clear light yellow
*V* = 805.71 (12) Å^3^	0.45 × 0.4 × 0.2 mm

### Bis(µ-3-methylpyridine N-oxide)bis[aquadibromido(3-methylpyridine N-oxide)manganese(II)] (III) Data collection

**Table:**

Rigaku XtaLAB mini diffractometer	3672 independent reflections
Radiation source: fine-focus sealed X-ray tube, Enhance (Mo) X-ray Source	2875 reflections with *I* > 2σ(*I*)
Graphite Monochromator monochromator	*R*_int_ = 0.036
Detector resolution: 13.6612 pixels mm^-1^	θ_max_ = 27.5°, θ_min_ = 1.9°
profile data from ω–scans	*h* = −9→9
Absorption correction: multi-scan (CrysAlisPro; Rigaku Oxford Diffraction, 2018)	*k* = −12→12
*T*_min_ = 0.319, *T*_max_ = 1.000	*l* = −15→15
8460 measured reflections	

### Bis(µ-3-methylpyridine N-oxide)bis[aquadibromido(3-methylpyridine N-oxide)manganese(II)] (III) Refinement

**Table:**

Refinement on *F*^2^	Primary atom site location: dual
Least-squares matrix: full	Hydrogen site location: mixed
*R*[*F*^2^ > 2σ(*F*^2^)] = 0.040	H atoms treated by a mixture of independent and constrained refinement
*wR*(*F*^2^) = 0.098	*w* = 1/[σ^2^(*F*_o_^2^) + (0.0461*P*)^2^ + 0.5922*P*] where *P* = (*F*_o_^2^ + 2*F*_c_^2^)/3
*S* = 1.01	(Δ/σ)_max_ < 0.001
3672 reflections	Δρ_max_ = 1.66 e Å^−^^3^
191 parameters	Δρ_min_ = −0.84 e Å^−^^3^
2 restraints	

### Bis(µ-3-methylpyridine N-oxide)bis[aquadibromido(3-methylpyridine N-oxide)manganese(II)] (III) Special details

**Table:**

Geometry. All esds (except the esd in the dihedral angle between two l.s. planes) are estimated using the full covariance matrix. The cell esds are taken into account individually in the estimation of esds in distances, angles and torsion angles; correlations between esds in cell parameters are only used when they are defined by crystal symmetry. An approximate (isotropic) treatment of cell esds is used for estimating esds involving l.s. planes.

### Bis(µ-3-methylpyridine N-oxide)bis[aquadibromido(3-methylpyridine N-oxide)manganese(II)] (III) Fractional atomic coordinates and isotropic or equivalent isotropic displacement parameters (Å^2^)

**Table:**

	*x*	*y*	*z*	*U*_iso_*/*U*_eq_	
Br1	0.36280 (5)	0.42105 (5)	0.13651 (4)	0.03458 (13)	
C1	0.4906 (7)	0.7816 (7)	0.4116 (5)	0.0544 (14)	
H1	0.509132	0.827435	0.356286	0.065*	
N1	0.3196 (5)	0.7177 (4)	0.4046 (3)	0.0383 (9)	
Mn1	0.04801 (8)	0.51611 (7)	0.16098 (5)	0.02659 (16)	
O1	0.1816 (4)	0.7175 (4)	0.3187 (3)	0.0418 (8)	
O2	0.0827 (3)	0.6326 (3)	0.0380 (3)	0.0295 (6)	
C2	0.6381 (7)	0.7822 (7)	0.4963 (5)	0.0606 (16)	
Br2	−0.07392 (6)	0.33641 (5)	0.25163 (4)	0.03571 (14)	
N2	0.1876 (4)	0.7681 (4)	0.0757 (3)	0.0254 (7)	
C3	0.6062 (7)	0.7148 (6)	0.5770 (5)	0.0520 (13)	
H3	0.705412	0.710037	0.635284	0.062*	
O3	−0.2115 (4)	0.5986 (4)	0.1560 (3)	0.0360 (7)	
H3A	−0.304 (4)	0.546 (5)	0.159 (4)	0.045 (15)*	
H3B	−0.237 (7)	0.594 (6)	0.082 (3)	0.065 (18)*	
C4	0.4293 (7)	0.6553 (6)	0.5712 (5)	0.0489 (12)	
H4	0.406617	0.612501	0.627790	0.059*	
C5	0.2857 (6)	0.6573 (5)	0.4846 (4)	0.0391 (10)	
H5	0.163976	0.616659	0.481113	0.047*	
C6	0.8283 (9)	0.8554 (11)	0.5025 (7)	0.110 (3)	
H6A	0.848206	0.962978	0.548807	0.165*	
H6B	0.841882	0.833305	0.417405	0.165*	
H6C	0.918146	0.817490	0.545184	0.165*	
C7	0.3693 (5)	0.7876 (4)	0.1021 (4)	0.0287 (9)	
H7	0.422698	0.704972	0.095217	0.034*	
C8	0.4801 (5)	0.9247 (5)	0.1391 (4)	0.0317 (9)	
C9	0.3993 (6)	1.0418 (5)	0.1454 (4)	0.0369 (10)	
H9	0.472406	1.136963	0.166642	0.044*	
C10	0.2119 (6)	1.0206 (5)	0.1206 (5)	0.0425 (11)	
H10	0.155519	1.101679	0.127690	0.051*	
C11	0.1069 (6)	0.8811 (5)	0.0856 (4)	0.0376 (10)	
H11	−0.022312	0.865462	0.068630	0.045*	
C12	0.6838 (6)	0.9463 (6)	0.1743 (6)	0.0583 (15)	
H12A	0.727982	0.988271	0.265702	0.087*	
H12B	0.739282	1.014307	0.142149	0.087*	
H12C	0.717356	0.850553	0.138142	0.087*	

### Bis(µ-3-methylpyridine N-oxide)bis[aquadibromido(3-methylpyridine N-oxide)manganese(II)] (III) Atomic displacement parameters (Å^2^)

**Table:**

	*U*^11^	*U*^22^	*U*^33^	*U*^12^	*U*^13^	*U*^23^
Br1	0.0214 (2)	0.0436 (3)	0.0499 (3)	0.00744 (18)	0.00730 (17)	0.0319 (2)
C1	0.044 (3)	0.076 (4)	0.044 (3)	−0.004 (3)	0.009 (2)	0.031 (3)
N1	0.036 (2)	0.043 (2)	0.032 (2)	0.0035 (18)	0.0039 (16)	0.0153 (18)
Mn1	0.0209 (3)	0.0312 (3)	0.0304 (3)	−0.0003 (2)	0.0023 (2)	0.0195 (3)
O1	0.0405 (18)	0.0424 (19)	0.0391 (18)	0.0021 (15)	−0.0031 (14)	0.0214 (16)
O2	0.0230 (13)	0.0309 (15)	0.0352 (16)	−0.0048 (12)	0.0013 (11)	0.0207 (13)
C2	0.041 (3)	0.084 (4)	0.044 (3)	0.003 (3)	0.006 (2)	0.017 (3)
Br2	0.0330 (2)	0.0405 (3)	0.0419 (3)	0.00105 (19)	0.00990 (18)	0.0280 (2)
N2	0.0225 (16)	0.0287 (18)	0.0282 (17)	0.0018 (14)	0.0054 (13)	0.0169 (15)
C3	0.046 (3)	0.064 (4)	0.035 (3)	0.012 (3)	0.002 (2)	0.011 (3)
O3	0.0261 (16)	0.0437 (19)	0.047 (2)	0.0063 (14)	0.0113 (14)	0.0273 (17)
C4	0.051 (3)	0.057 (3)	0.039 (3)	0.009 (3)	0.008 (2)	0.022 (3)
C5	0.041 (2)	0.043 (3)	0.032 (2)	0.003 (2)	0.0093 (19)	0.015 (2)
C6	0.043 (4)	0.180 (9)	0.095 (6)	−0.025 (5)	0.007 (3)	0.064 (6)
C7	0.0226 (19)	0.025 (2)	0.040 (2)	0.0041 (17)	0.0054 (17)	0.0168 (19)
C8	0.028 (2)	0.029 (2)	0.040 (2)	0.0042 (18)	0.0103 (18)	0.017 (2)
C9	0.044 (3)	0.026 (2)	0.037 (2)	0.000 (2)	0.006 (2)	0.014 (2)
C10	0.045 (3)	0.028 (2)	0.055 (3)	0.013 (2)	0.005 (2)	0.019 (2)
C11	0.029 (2)	0.043 (3)	0.048 (3)	0.016 (2)	0.0118 (19)	0.023 (2)
C12	0.029 (2)	0.048 (3)	0.100 (5)	0.002 (2)	0.017 (3)	0.034 (3)

### Bis(µ-3-methylpyridine N-oxide)bis[aquadibromido(3-methylpyridine N-oxide)manganese(II)] (III) Geometric parameters (Å, º)

**Table:**

Mn1—Br1	2.7237 (7)	O3—H3B	0.844 (19)
C1—H1	0.9500	C4—H4	0.9500
C1—N1	1.347 (6)	C4—C5	1.370 (6)
C1—C2	1.368 (7)	C5—H5	0.9500
N1—O1	1.328 (4)	C6—H6A	0.9800
N1—C5	1.346 (6)	C6—H6B	0.9800
Mn1—O1	2.129 (3)	C6—H6C	0.9800
Mn1—O2	2.211 (3)	C7—H7	0.9500
Mn1—O2^i^	2.219 (3)	C7—C8	1.372 (6)
Mn1—Br2	2.5687 (7)	C8—C9	1.377 (6)
Mn1—O3	2.245 (3)	C8—C12	1.499 (6)
O2—N2	1.339 (4)	C9—H9	0.9500
C2—C3	1.399 (8)	C9—C10	1.377 (6)
C2—C6	1.511 (8)	C10—H10	0.9500
N2—C7	1.336 (5)	C10—C11	1.377 (6)
N2—C11	1.332 (5)	C11—H11	0.9500
C3—H3	0.9500	C12—H12A	0.9800
C3—C4	1.379 (7)	C12—H12B	0.9800
O3—H3A	0.834 (19)	C12—H12C	0.9800
			
N1—C1—H1	119.2	Mn1—O3—H3B	101 (4)
N1—C1—C2	121.6 (5)	H3A—O3—H3B	104 (5)
C2—C1—H1	119.2	C3—C4—H4	119.7
O1—N1—C1	119.2 (4)	C5—C4—C3	120.7 (5)
O1—N1—C5	119.5 (4)	C5—C4—H4	119.7
C5—N1—C1	121.3 (4)	N1—C5—C4	119.1 (4)
O1—Mn1—Br1	93.86 (9)	N1—C5—H5	120.4
O1—Mn1—O2^i^	157.76 (11)	C4—C5—H5	120.4
O1—Mn1—O2	88.94 (11)	C2—C6—H6A	109.5
O1—Mn1—Br2	105.48 (9)	C2—C6—H6B	109.5
O1—Mn1—O3	88.95 (12)	C2—C6—H6C	109.5
O2—Mn1—Br1	91.19 (7)	H6A—C6—H6B	109.5
O2^i^—Mn1—Br1	89.84 (8)	H6A—C6—H6C	109.5
O2—Mn1—O2^i^	69.05 (11)	H6B—C6—H6C	109.5
O2^i^—Mn1—Br2	95.91 (7)	N2—C7—H7	119.4
O2—Mn1—Br2	163.39 (7)	N2—C7—C8	121.2 (4)
O2—Mn1—O3	81.25 (11)	C8—C7—H7	119.4
O2^i^—Mn1—O3	84.75 (11)	C7—C8—C9	118.3 (4)
Br2—Mn1—Br1	95.91 (2)	C7—C8—C12	120.6 (4)
O3—Mn1—Br1	171.89 (8)	C9—C8—C12	121.1 (4)
O3—Mn1—Br2	90.65 (8)	C8—C9—H9	120.1
N1—O1—Mn1	119.7 (3)	C8—C9—C10	119.8 (4)
Mn1—O2—Mn1^i^	110.95 (11)	C10—C9—H9	120.1
N2—O2—Mn1^i^	123.8 (2)	C9—C10—H10	120.2
N2—O2—Mn1	124.7 (2)	C11—C10—C9	119.6 (4)
C1—C2—C3	117.8 (5)	C11—C10—H10	120.2
C1—C2—C6	120.5 (6)	N2—C11—C10	119.6 (4)
C3—C2—C6	121.7 (5)	N2—C11—H11	120.2
C7—N2—O2	119.9 (3)	C10—C11—H11	120.2
C11—N2—O2	118.7 (3)	C8—C12—H12A	109.5
C11—N2—C7	121.5 (4)	C8—C12—H12B	109.5
C2—C3—H3	120.3	C8—C12—H12C	109.5
C4—C3—C2	119.4 (5)	H12A—C12—H12B	109.5
C4—C3—H3	120.3	H12A—C12—H12C	109.5
Mn1—O3—H3A	118 (3)	H12B—C12—H12C	109.5

Symmetry code: (i) −*x*, −*y*+1, −*z*.

### Bis(µ-3-methylpyridine N-oxide)bis[aquadibromido(3-methylpyridine N-oxide)manganese(II)] (III) Hydrogen-bond geometry (Å, º)

**Table:**

*D*—H···*A*	*D*—H	H···*A*	*D*···*A*	*D*—H···*A*
O3—H3*A*···Br1^ii^	0.83 (2)	2.60 (2)	3.410 (3)	164 (4)
O3—H3*B*···Br1^i^	0.84 (2)	2.55 (2)	3.386 (3)	172 (5)

Symmetry codes: (i) −*x*, −*y*+1, −*z*; (ii) *x*−1, *y*, *z*.

### Bis(µ-4-methylpyridine N-oxide)bis[dibromidomethanol(4-methylpyridine N-oxide)manganese(II)] (IV) Crystal data

**Table:**

[Mn_2_Br_4_(C_6_H_7_NO)_4_(CH_4_O)_2_]	*F*(000) = 916
*M**_r_* = 930.11	*D*_x_ = 1.790 Mg m^−^^3^
Monoclinic, *P*2_1_/*n*	Mo *K*α radiation, λ = 0.71073 Å
*a* = 13.5384 (7) Å	Cell parameters from 9251 reflections
*b* = 9.5354 (4) Å	θ = 2.0–33.1°
*c* = 13.7292 (7) Å	µ = 5.40 mm^−^^1^
β = 103.112 (5)°	*T* = 170 K
*V* = 1726.15 (15) Å^3^	Prism, clear light brown
*Z* = 2	0.4 × 0.4 × 0.4 mm

### Bis(µ-4-methylpyridine N-oxide)bis[dibromidomethanol(4-methylpyridine N-oxide)manganese(II)] (IV) Data collection

**Table:**

Rigaku XtaLAB mini diffractometer	3964 independent reflections
Radiation source: fine-focus sealed X-ray tube, Enhance (Mo) X-ray Source	3175 reflections with *I* > 2σ(*I*)
Graphite Monochromator monochromator	*R*_int_ = 0.061
Detector resolution: 13.6612 pixels mm^-1^	θ_max_ = 27.5°, θ_min_ = 1.9°
profile data from ω–scans	*h* = −17→17
Absorption correction: multi-scan (CrysAlisPro; Rigaku Oxford Diffraction, 2018)	*k* = −12→12
*T*_min_ = 0.659, *T*_max_ = 1.000	*l* = −17→17
17754 measured reflections	

### Bis(µ-4-methylpyridine N-oxide)bis[dibromidomethanol(4-methylpyridine N-oxide)manganese(II)] (IV) Refinement

**Table:**

Refinement on *F*^2^	Primary atom site location: dual
Least-squares matrix: full	Hydrogen site location: mixed
*R*[*F*^2^ > 2σ(*F*^2^)] = 0.043	H atoms treated by a mixture of independent and constrained refinement
*wR*(*F*^2^) = 0.112	*w* = 1/[σ^2^(*F*_o_^2^) + (0.0665*P*)^2^ + 0.0317*P*] where *P* = (*F*_o_^2^ + 2*F*_c_^2^)/3
*S* = 1.03	(Δ/σ)_max_ = 0.001
3964 reflections	Δρ_max_ = 1.64 e Å^−^^3^
196 parameters	Δρ_min_ = −0.74 e Å^−^^3^
3 restraints	

### Bis(µ-4-methylpyridine N-oxide)bis[dibromidomethanol(4-methylpyridine N-oxide)manganese(II)] (IV) Special details

**Table:**

Geometry. All esds (except the esd in the dihedral angle between two l.s. planes) are estimated using the full covariance matrix. The cell esds are taken into account individually in the estimation of esds in distances, angles and torsion angles; correlations between esds in cell parameters are only used when they are defined by crystal symmetry. An approximate (isotropic) treatment of cell esds is used for estimating esds involving l.s. planes.

### Bis(µ-4-methylpyridine N-oxide)bis[dibromidomethanol(4-methylpyridine N-oxide)manganese(II)] (IV) Fractional atomic coordinates and isotropic or equivalent isotropic displacement parameters (Å^2^)

**Table:**

	*x*	*y*	*z*	*U*_iso_*/*U*_eq_	
Br1	0.55122 (3)	0.55895 (4)	0.73950 (3)	0.02951 (13)	
Mn1	0.42974 (4)	0.62247 (6)	0.55858 (4)	0.02011 (15)	
N1	0.4741 (3)	0.9181 (3)	0.6510 (2)	0.0243 (7)	
C1	0.4328 (3)	0.8828 (4)	0.7272 (3)	0.0266 (9)	
H1	0.390399	0.802474	0.722371	0.032*	
O1	0.4550 (2)	0.8416 (3)	0.56661 (19)	0.0283 (6)	
O2	0.4393 (2)	0.4023 (3)	0.5134 (2)	0.0219 (6)	
N2	0.3727 (2)	0.3013 (3)	0.5239 (2)	0.0209 (7)	
C2	0.4519 (3)	0.9632 (4)	0.8130 (3)	0.0275 (9)	
H2	0.422582	0.937169	0.866939	0.033*	
Br2	0.25806 (3)	0.60062 (4)	0.60914 (3)	0.03081 (13)	
C3	0.5128 (3)	1.0806 (4)	0.8220 (3)	0.0292 (9)	
O3	0.3548 (2)	0.6580 (3)	0.3987 (2)	0.0297 (6)	
H3	0.3793 (18)	0.613 (4)	0.3550 (13)	0.045*	
C4	0.5541 (3)	1.1132 (4)	0.7408 (3)	0.0279 (9)	
H4	0.596641	1.193074	0.743893	0.033*	
C5	0.5341 (3)	1.0312 (4)	0.6560 (3)	0.0269 (9)	
H5	0.562828	1.054615	0.601121	0.032*	
C6	0.5321 (4)	1.1692 (5)	0.9149 (3)	0.0417 (11)	
H6A	0.476217	1.157376	0.948694	0.063*	
H6B	0.536834	1.267992	0.896711	0.063*	
H6C	0.595831	1.140083	0.959757	0.063*	
C7	0.3259 (3)	0.2277 (4)	0.4442 (3)	0.0256 (8)	
H7	0.339610	0.246866	0.380646	0.031*	
C8	0.2582 (3)	0.1247 (4)	0.4535 (3)	0.0305 (9)	
H8	0.226450	0.071416	0.396473	0.037*	
C9	0.2353 (3)	0.0971 (4)	0.5451 (4)	0.0318 (10)	
C10	0.2848 (3)	0.1774 (4)	0.6253 (3)	0.0294 (9)	
H10	0.270434	0.162602	0.689092	0.035*	
C11	0.3542 (3)	0.2781 (4)	0.6148 (3)	0.0255 (8)	
H11	0.388719	0.330637	0.671008	0.031*	
C12	0.1594 (4)	−0.0123 (5)	0.5565 (4)	0.0475 (13)	
H12A	0.105685	−0.016658	0.495319	0.071*	
H12B	0.129842	0.011844	0.613227	0.071*	
H12C	0.193117	−0.103673	0.568581	0.071*	
C13	0.2496 (4)	0.6767 (8)	0.3550 (4)	0.069 (2)	
H13A	0.241626	0.735075	0.294999	0.103*	
H13B	0.216868	0.722797	0.403254	0.103*	
H13C	0.217904	0.585124	0.336828	0.103*	

### Bis(µ-4-methylpyridine N-oxide)bis[dibromidomethanol(4-methylpyridine N-oxide)manganese(II)] (IV) Atomic displacement parameters (Å^2^)

**Table:**

	*U*^11^	*U*^22^	*U*^33^	*U*^12^	*U*^13^	*U*^23^
Br1	0.0332 (2)	0.0303 (2)	0.0229 (2)	0.00096 (17)	0.00200 (17)	−0.00194 (16)
Mn1	0.0218 (3)	0.0170 (3)	0.0229 (3)	−0.0003 (2)	0.0080 (2)	−0.0004 (2)
N1	0.0301 (18)	0.0184 (16)	0.0254 (17)	0.0024 (13)	0.0085 (15)	−0.0013 (13)
C1	0.032 (2)	0.021 (2)	0.030 (2)	−0.0061 (16)	0.0127 (19)	−0.0017 (16)
O1	0.0437 (17)	0.0190 (14)	0.0245 (14)	−0.0034 (12)	0.0125 (13)	−0.0045 (11)
O2	0.0236 (13)	0.0171 (13)	0.0271 (14)	−0.0053 (10)	0.0103 (12)	−0.0032 (11)
N2	0.0189 (15)	0.0168 (16)	0.0291 (17)	0.0001 (12)	0.0098 (14)	0.0013 (13)
C2	0.031 (2)	0.029 (2)	0.025 (2)	0.0034 (17)	0.0130 (18)	0.0010 (17)
Br2	0.0271 (2)	0.0298 (2)	0.0401 (3)	−0.00056 (16)	0.01705 (19)	−0.00008 (18)
C3	0.027 (2)	0.030 (2)	0.030 (2)	0.0036 (17)	0.0038 (18)	−0.0032 (17)
O3	0.0291 (15)	0.0340 (16)	0.0247 (14)	0.0083 (13)	0.0035 (13)	−0.0007 (12)
C4	0.029 (2)	0.024 (2)	0.031 (2)	−0.0017 (16)	0.0069 (18)	0.0002 (16)
C5	0.030 (2)	0.023 (2)	0.029 (2)	0.0012 (17)	0.0090 (18)	0.0051 (16)
C6	0.047 (3)	0.043 (3)	0.035 (2)	−0.010 (2)	0.008 (2)	−0.009 (2)
C7	0.025 (2)	0.027 (2)	0.0239 (19)	−0.0003 (16)	0.0035 (17)	−0.0041 (16)
C8	0.027 (2)	0.024 (2)	0.038 (2)	−0.0039 (17)	0.0029 (19)	−0.0064 (18)
C9	0.023 (2)	0.020 (2)	0.052 (3)	0.0039 (16)	0.009 (2)	0.0052 (19)
C10	0.034 (2)	0.023 (2)	0.033 (2)	0.0035 (17)	0.0102 (19)	0.0090 (17)
C11	0.030 (2)	0.023 (2)	0.0239 (18)	0.0032 (16)	0.0065 (17)	0.0020 (16)
C12	0.044 (3)	0.031 (3)	0.068 (4)	−0.009 (2)	0.015 (3)	0.010 (2)
C13	0.030 (3)	0.136 (6)	0.037 (3)	0.012 (3)	−0.001 (2)	0.014 (3)

### Bis(µ-4-methylpyridine N-oxide)bis[dibromidomethanol(4-methylpyridine N-oxide)manganese(II)] (IV) Geometric parameters (Å, º)

**Table:**

Mn1—Br1	2.7181 (7)	C4—C5	1.377 (6)
Mn1—O1	2.116 (3)	C5—H5	0.9500
Mn1—O2	2.201 (2)	C6—H6A	0.9800
Mn1—O2^i^	2.230 (3)	C6—H6B	0.9800
Mn1—Br2	2.5806 (7)	C6—H6C	0.9800
Mn1—O3	2.225 (3)	C7—H7	0.9500
N1—C1	1.336 (5)	C7—C8	1.370 (6)
N1—O1	1.344 (4)	C8—H8	0.9500
N1—C5	1.343 (5)	C8—C9	1.387 (6)
C1—H1	0.9500	C9—C10	1.382 (6)
C1—C2	1.379 (6)	C9—C12	1.497 (6)
O2—N2	1.349 (4)	C10—H10	0.9500
N2—C7	1.333 (5)	C10—C11	1.373 (5)
N2—C11	1.345 (5)	C11—H11	0.9500
C2—H2	0.9500	C12—H12A	0.9800
C2—C3	1.379 (6)	C12—H12B	0.9800
C3—C4	1.390 (6)	C12—H12C	0.9800
C3—C6	1.502 (6)	C13—H13A	0.9800
O3—H3	0.861 (9)	C13—H13B	0.9800
O3—C13	1.426 (6)	C13—H13C	0.9800
C4—H4	0.9500		
			
O1—Mn1—Br1	96.62 (8)	C5—C4—C3	120.9 (4)
O1—Mn1—O2	159.59 (11)	C5—C4—H4	119.6
O1—Mn1—O2^i^	89.55 (10)	N1—C5—C4	120.0 (4)
O1—Mn1—Br2	102.17 (8)	N1—C5—H5	120.0
O1—Mn1—O3	86.17 (11)	C4—C5—H5	120.0
O2^i^—Mn1—Br1	90.24 (7)	C3—C6—H6A	109.5
O2—Mn1—Br1	89.03 (7)	C3—C6—H6B	109.5
O2—Mn1—O2^i^	70.77 (11)	C3—C6—H6C	109.5
O2—Mn1—Br2	96.47 (7)	H6A—C6—H6B	109.5
O2^i^—Mn1—Br2	165.02 (7)	H6A—C6—H6C	109.5
O2—Mn1—O3	84.83 (10)	H6B—C6—H6C	109.5
Br2—Mn1—Br1	97.55 (2)	N2—C7—H7	119.8
O3—Mn1—Br1	168.90 (8)	N2—C7—C8	120.3 (4)
O3—Mn1—O2^i^	79.01 (10)	C8—C7—H7	119.8
O3—Mn1—Br2	92.34 (8)	C7—C8—H8	119.6
C1—N1—O1	120.4 (3)	C7—C8—C9	120.8 (4)
C1—N1—C5	121.2 (3)	C9—C8—H8	119.6
C5—N1—O1	118.4 (3)	C8—C9—C12	121.8 (4)
N1—C1—H1	120.1	C10—C9—C8	116.7 (4)
N1—C1—C2	119.8 (4)	C10—C9—C12	121.5 (4)
C2—C1—H1	120.1	C9—C10—H10	119.2
N1—O1—Mn1	125.3 (2)	C11—C10—C9	121.5 (4)
Mn1—O2—Mn1^i^	109.23 (11)	C11—C10—H10	119.2
N2—O2—Mn1^i^	126.1 (2)	N2—C11—C10	119.2 (4)
N2—O2—Mn1	124.6 (2)	N2—C11—H11	120.4
C7—N2—O2	119.5 (3)	C10—C11—H11	120.4
C7—N2—C11	121.4 (3)	C9—C12—H12A	109.5
C11—N2—O2	119.1 (3)	C9—C12—H12B	109.5
C1—C2—H2	119.3	C9—C12—H12C	109.5
C3—C2—C1	121.4 (4)	H12A—C12—H12B	109.5
C3—C2—H2	119.3	H12A—C12—H12C	109.5
C2—C3—C4	116.8 (4)	H12B—C12—H12C	109.5
C2—C3—C6	121.2 (4)	O3—C13—H13A	109.5
C4—C3—C6	122.0 (4)	O3—C13—H13B	109.5
Mn1—O3—H3	116.8 (15)	O3—C13—H13C	109.5
C13—O3—Mn1	128.5 (3)	H13A—C13—H13B	109.5
C13—O3—H3	105.9 (15)	H13A—C13—H13C	109.5
C3—C4—H4	119.6	H13B—C13—H13C	109.5

Symmetry code: (i) −*x*+1, −*y*+1, −*z*+1.

### Bis(µ-4-methylpyridine N-oxide)bis[dibromidomethanol(4-methylpyridine N-oxide)manganese(II)] (IV) Hydrogen-bond geometry (Å, º)

**Table:**

*D*—H···*A*	*D*—H	H···*A*	*D*···*A*	*D*—H···*A*
O3—H3···Br1^i^	0.86 (1)	2.41 (2)	3.255 (3)	166 (3)

Symmetry code: (i) −*x*+1, −*y*+1, −*z*+1.
